# Supplementary material for: Analysis of Tonsil Tissues from Patients Diagnosed with Chronic Tonsillitis—Microbiological Profile, Biofilm-Forming Capacity and Histology
Source: Antibiotics (Basel). 2022 Dec 3;11(12):1747. doi: 10.3390/antibiotics11121747 (PMC9774359; doi:10.3390/antibiotics11121747)
Supplement: Supplementary file 1 [file antibiotics-11-01747-s001.zip › antibiotics-2034858-supplementary.pdf]

Supplementary File

**Table S1.** MALDI-ToF data for bacteria isolated from tonsillar tissue from patients with chronic tonsilitis (Group I and Group II).

| | Code name | Specimen name | Confidence value (%) |
|---|---|---|---|
| **Group I** (isolates from palatine tissue) | oT_001_16 | *Streptococcus oralis* | 99.9 |
| | oT_002_16 | *Streptococcus dysgalactiae* | 99.9 |
| | oT_002_16 | *Streptococcus pyogenes* | 99.9 |
| | oT_003_16 | *Streptococcus parasanguinis* | 99.9 |
| | oT_001_17 | *Streptococcus parasanguinis* | 99.9 |
| | oT_003_17 | *Streptococcus parasanguinis* | 99.9 |
| | oT_004_17 | *Staphylococcus aureus* | 99.9 |
| | oT_005_17 | *Streptococcus oralis* | 99.9 |
| | oT_006_17 | *Staphylococcus aureus* | 99.9 |
| | oT_007_17 | *Streptococcus oralis* | 99.9 |
| | oT_007_17 | *Staphylococcus warneri* | 99.9 |
| | oT_010_17 | *Streptococcus parasanguinis* | 99.9 |
| | oT_011_17 | *Staphylococcus aureus* | 99.9 |
| | oT_011_17 | *Streptococcus oralis* | 99.9 |
| | oT_011_17 | *Rothia mucilaginosa* | 99.9 |
| | oT_012_17 | *Streptococcus oralis* | 99.9 |
| | oT_013_17 | *Staphylococcus aureus* | 99.9 |
| | oT_014_17 | *Staphylococcus hominis* | 99.9 |
| | oT_014_17 | *Rothia mucilaginosa* | 99.9 |
| | oT_015_17 | *Staphylococcus aureus* | 99.9 |
| | oT_017_17 | *Enterobacter cloacae* | 99.9 |
| | oT_018_17 | *Enterobacter cloacae* | 99.9 |
| | oT_019_17 | *Staphylococcus aureus* | 99.9 |
| | oT_020_17 | *Streptococcus agalactiae* | 99.9 |
| | oT_020_17 | *Streptococcus oralis* | 99.9 |
| | oT_021_17 | *Staphylococcus aureus* | 99.9 |
| | oT_021_17 | *Streptococcus dysgalactiae* | 99.9 |
| | oT_022_17 | *Streptococcus oralis* | 99.9 |
| | oT_022_17 | *Rothia mucilaginosa* | 99.9 |
| | oT_023_17 | *Staphylococcus aureus* | 99.9 |
| | oT_024_17 | *Streptococcus parasanguinis* | 99.9 |
| | oT_025_17 | *Staphylococcus aureus* | 99.9 |
| | oT_026_17 | *Streptococcus anginosus* | 99.9 |
| | oT_027_17 | *Streptococcus parasanguinis* | 99.9 |
| | oT_028_17 | *Staphylococcus aureus* | 99.9 |
| **Group II** (isolates from palatine tissue) | dT_003_16 | *Streptococcus oralis* | 99.9 |
| | dT_001_17 | *Streptococcus consellatus* | 99.9 |
| | dT_001_17 | *Streptococcus angiosus* | 99.9 |
| | dT_002_17 | *Streptococcus parasanguinis* | 99.9 |
| | dT_003_17 | *Streptococcus oralis* | 99.9 |

| | | | |
|---|---|---|---|
| | dT_004_17 | *Streptococcus oralis* | 99.9 |
| | dT_005_17 | *Streptococcus oralis* | 99.9 |
| | dT_006_17 | *Staphylococcus aureus* | 99.9 |
| | dT_007_17 | *Streptococcus oralis* | 99.9 |
| | dT_009_17 | *Streptococcus parasanguinis* | 99.9 |
| | dT_009_17 | *Micrococcus luteus* | 99.9 |
| | dT_010_17 | *Streptococcus oralis* | 99.9 |
| | dT_011_17 | *Streptococcus oralis* | 99.9 |
| | dT_011_17 | *Staphylococcus aureus* | 99.9 |
| | dT_012_17 | *Streptococcus salivarius* | 99.9 |
| | dT_013_17 | *Streptococcus parasanguinis* | 99.9 |
| | dT_014_17 | *Streptococcus pyogenes* | 99.9 |
| | dT_015_17 | *Streptococcus pyogenes* | 99.9 |
| | dT_016_17 | *Streptococcus oralis* | 99.9 |
| | dT_017_17 | *Streptococcus parasanguinis* | 99.9 |
| | dT_019_17 | *Streptococcus oralis* | 99.9 |
| | dT_020_17 | *Staphylococcus aureus* | 99.9 |
| | dT_020_17 | *Streptococcus oralis* | 99.9 |
| | dT_022_17 | *Streptococcus consellatus* | 99.9 |
| | dT_022_17 | *Streptococcus pseudopneumoniae* | 99.9 |
| | dT_023_17 | *Streptococcus oralis* | 99.9 |
| | dT_024_17 | *Streptococcus oralis* | 99.9 |
| | dT_025_17 | *Streptococcus oralis* | 99.9 |
| | dT_025_17 | *Streptococcus aureus* | 99.9 |
| | dT_026_17 | *Streptococcus parasanguinis* | 99.9 |
| | dT_027_17 | *Streptococcus parasanguinis* | 99.9 |
| | dT_027_17 | *Streptococcus oralis* | 99.9 |
| | dT_028_17 | *Staphylococcus aureus* | 99.9 |
| **Group II** (isolates from adenoid tissue) | a_001_17 | *Streptococcus oralis* | 99.9 |
| | a_003_17 | *Streptococcus parasanguinis* | 99.9 |
| | a_005_17 | *Staphylococcus aureus* | 99.9 |
| | a_006_17 | *Streptococcus parasanguinis* | 99.9 |
| | a_007_17 | *Streptococcus parasanguinis* | 99.9 |
| | a_008_17 | *Streptococcus oralis* | 99.9 |
| | a_009_17 | *Staphylococcus aureus* | 99.9 |
| | a_010_17 | *Streptococcus oralis* | 99.9 |
| | a_011_17 | *Streptococcus oralis* | 99.9 |
| | a_012_17 | *Streptococcus oralis* | 99.9 |
| | a_013_17 | *Stenotrophomonas maltophilia* | 99.9 |
| | a_014_17 | *Streptococcus pyogenes* | 99.9 |
| | a_015_17 | *Streptococcus parasanguinis* | 99.9 |
| | a_015_17 | *Streptococcus agalactiae* | 99.9 |

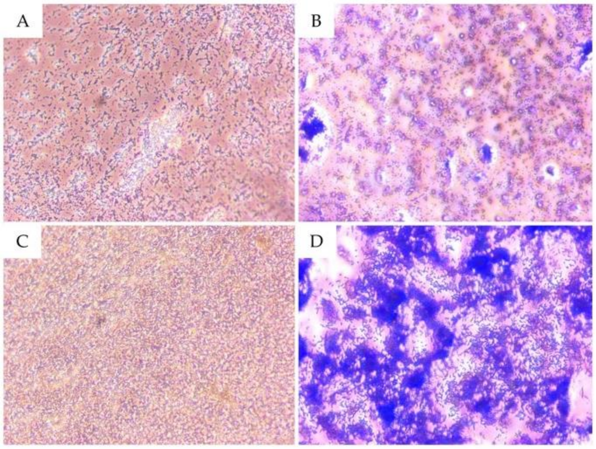


**Figure S1.** Biofilm formation of two *S. aureus* isolates from tonsilar tissue. (**A**) *S. aureus* isolate with moderate biofilm formation ability after washing (10X); (**B**) *S. aureus* isolate with moderate biofilm formation activity after crystal violet staining (10X); (**C**) *S. aureus* isolate with strong biofilm formation ability after washing (10X); (**D**) *S. aureus* isolate with strong biofilm formation activity after crystal violet staining (10X). All images were taken with Nikon eclipse Ts2 (Nikon T2, Tokyo, Japan; BIB-100, Boeco, Germany).
